# Design, Synthesis, and Acute Toxicity Assays for Novel Thymoquinone Derivative TQFL12 in Mice and the Mechanism of Resistance to Toxicity

**DOI:** 10.3390/molecules28135149

**Published:** 2023-06-30

**Authors:** Ting Li, Qi Tan, Chunli Wei, Hui Zou, Xiaoyan Liu, Zhiqiang Mei, Pengfei Zhang, Jingliang Cheng, Junjiang Fu

**Affiliations:** 1 Key Laboratory of Epigenetics and Oncology, The Research Center for Preclinical Medicine, Southwest Medical University, Luzhou 646000, China; 20220199120034@stu.swmu.edu.cn (T.L.); 20210199120024@stu.swmu.edu.cn (Q.T.); weichunli2015@swmu.edu.cn (C.W.); zouhui@hunnu.edu.cn (H.Z.); jczxlxy@swmu.edu.cn (X.L.); meizhiqiang@swmu.edu.cn (Z.M.); jingliangc@swmu.edu.cn (J.C.); 2 Basic Medical School, Southwest Medical University, Luzhou 646000, China; 3 Key Laboratory of Study and Discovery of Small Targeted Molecules of Hunan Province, School of Medicine, Hunan Normal University, Changsha 410013, China; 4 NHC Key Laboratory of Cancer Proteomics, Department of Oncology, Central South University, Changsha 410008, China; zhangpf690421@csu.edu.cn

**Keywords:** TQ, TQFL12, toxicity, mouse, triple-negative breast cancer, anti-cancer, AMPKα

## Abstract

TQFL12 is a novel derivative designed and synthesized on the basis of Thymoquinone (TQ) which is extracted from *Nigella sativa* seeds. We have demonstrated that TQFL12 was more effective in the treatment of TNBC than TQ. In order to directly reflect the acute toxicity of TQFL12 in vivo, in this study, we designed, synthesized, and compared it with TQ. The mice were administered drugs with different concentration gradients intraperitoneally, and death was observed within one week. The 24 h median lethal dose (LD_50_) of TQ was calculated to be 33.758 mg/kg, while that of TQFL12 on the 7th day was 81.405 mg/kg, and the toxicity was significantly lower than that of TQ. The liver and kidney tissues of the dead mice were observed by H&E staining. The kidneys of the TQ group had more severe renal damage, while the degree of the changes in the TQFL12 group was obviously less than that in the TQ group. Western blotting results showed that the expressions of phosphorylated levels of adenylate-activated protein kinase AMPKα were significantly up-regulated in the kidneys of the TQFL12 group. Therefore, it can be concluded that the acute toxicity of TQFL12 in vivo is significantly lower than that of TQ, and its anti-toxicity mechanism may be carried out through the AMPK signaling pathway, which has a good prospect for drug development.

## 1. Introduction

Malignant tumors are a worldwide disease that seriously threaten human health with tumor metastasis and recurrence. The incidence and mortality of tumors compared to chronic diseases are increasing year by year. The latest cancer data shows that breast cancer (BC) has become the number one killer of females due to malignant tumors in the world today [1,2,3,4]. Both the incidence and mortality of breast cancer are the top among female malignant tumors in many countries [5]. Triple-negative breast cancer (TNBC), as a result of lacking estrogen receptor (ERα), progesterone receptor (PR), and human epidermal growth factor receptor 2 (HER2), leads to high aggressiveness, strong metastasis, and drug resistance to traditional treatments, and has always attracted much attention in the fields of tumor research [6,7]. Poor diagnostic targets and a lack of effective therapeutic agents are the most two difficult problems. Among the different tumor treatment options including radiotherapy, chemotherapy, biologically targeted therapy, immunotherapy, and endocrine therapy, small-molecule compound therapy is considered to be highly effective and promising [8]. The need to find more highly effective with low toxicity therapeutic drugs targeting breast cancer, especially triple-negative breast cancer, has become urgent.

Natural products or traditional herbal medicine have long been the source of active ingredients for clinical therapeutics. Artemisinin (Qinghaosu) is a source of traditional Chinese herbal medicine with antimalarial effects found by Youyou Tu, for example [9]. Degalactotigonin is a Natural Compound from *Solanum nigrum* L., which has been demonstrated to inhibit the growth and metastasis of osteosarcoma [10]. Xianhe Bai et al. reported that Honokiol inhibits angiogenesis in vitro and tumor growth in vivo as a small-molecular-weight natural product [11]. However, the reasons why they have always not been widely accepted were the limited efficacy and unclear mechanisms [10,11,12]. In the study of Youyou Tu, they found that dihydroartemisinin was more stable and more effective than artemisinin; also, there was much less recurrence during treatment with this derivative [9].

Interestingly, Thymoquinone (TQ) is also an active small-molecule compound extracted from seeds of the natural product *Nigella sativa* that has been reported to be efficacious for a great number of diseases including cancer, inflammatory and immunological diseases, and so on [13,14]. Particularly, TQ has been reported as inhibiting the proliferation and migration of breast cancer cells with promising prospects [15,16,17,18]. Zhang et al. reported that TQ has a strong cytotoxicity on bladder cancer cells because it can inhibit their proliferation and cause apoptosis [19]. Samarghandian et al. reported that TQ can induce antitumor effects and apoptosis in lung cancer cells [20]. However, TQ is a weak anticancer constituent of black seeds oil [21], and the effective drug concentration is as high as 165 µM for breast cancer cells [22]. TQ treatment with 50 and 100 µM doses can be found to suppress the growth of MDA-MB-231 breast tumor cells and tumor vascular volume. [23]. For that reason, it is necessary to obtain more-effective and lower-toxicity TQ derivatives.

In our previous study, we obtained a novel derivative TQFL12 of TQ. TQFL12 (molecular formula: C_17_H_16_ClNO_2_) has been demonstrated to inhibit cell growth, migration, and invasion of breast cancer in vitro, as well as tumor growth in vivo. We found that the suppression of the proliferative effects of TQFL12 against TNBC cells is stronger than TQ by conducting CCK8 assays in human TNBC cells (BT549 and MDA-MB-231) and mouse breast cancer cells (4T1) with TQ and TQFL12 at different times, and TQFL12 abolished different aspects of TNBC development, including cell proliferation, migration, invasion, and apoptosis. In addition, we also found that TQFL12 treatment not only inhibits cancer cell migration and invasion but also induces cell death. TQFL12 was demonstrated to slightly affect the cell cycle while significantly affecting cell apoptosis in 4T1 cells. As for the in vivo assays, we found TQFL12 suppressed tumor growth and metastasis in the 4T1 cell xenograft mouse model, with more effectivity compared with TQ, thus unambiguously indicating that TQFL12 is more capable of suppressing TNBC cell growth, migration, and invasion. Interestingly, we found that mice treated with TQFL12 showed more air bubbles compared to those of the control group in the tumor pathological changes [24]. Due to the effect of tumor suppression in mice, it is necessary to study its toxic effects in vivo.

## 2. Results

### 2.1. Design and Synthesis of TQFL12

**Figure 1:**
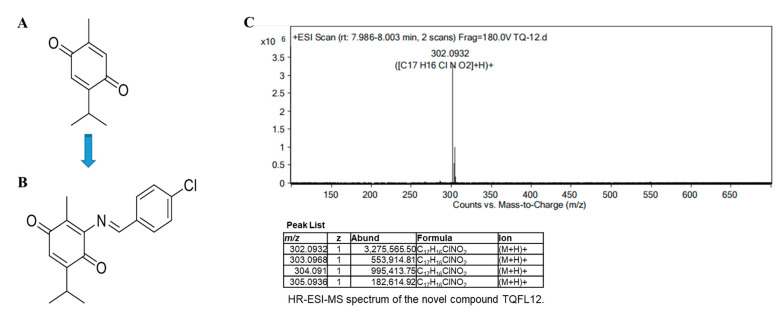
The design and synthesis of the novel compound TQFL12. The NH_2_ group using NaN_3_ and acetic acid and 4-chlorobenzaldehyd in EtOH with HCl were added on the TQ position 6. (**A**) The original structure of TQ. (**B**) The structure of the novel derivative TQFL12 [(E)-3-((4-chlorobenzylidene) amino)-5-isopropyl-2-methylcyclohexa-2,5-diene-1,4-dione]. (**C**) HR-ESI-MS spectrum of TQFL12. TQFL12 yields four different mass-to-charge ratios, with maximum abundance at 302.0932, and the relative molecular mass of TQFL12 is correctly verified to *m*/*z* 302.0932 [M + H]^+^ (calcd. for C_17_H_17_ClNO_2_^+^: 302.0932) after hydrogenation by the mass spectrometer.

To retain the quinone group in the structure of thymoquinone during the synthesis of TQ derivatives, we focus on TQ position 6 to explore the effect of different substituents on their activity [21]. We thus used two steps to synthesize TQFL12 successfully. The intermediate product 3- amino- 5- isopropyl- 2-methylcyclohexa- 2,5- diene- 1,4- dione (NTQ) was first synthesized by adding an NH_2_ group using NaN_3_ and acetic acid. Then, further synthesis of TQFL12 was finished by 4-chlorobenzaldehyd in EtOH with HCl (Figure 1A,B). The obtained TQFL12 was a yellow solid and yielded 85.1%. The results of HR-ESI-MS are shown in Figure 1C. After hydrogenation by the mass spectrometer, TQFL12 yielded four different mass-to-charge ratios as shown in the table of Figure 1C, with maximum abundance at 302.0932, and the relative molecular mass of TQFL12 is correctly verified to *m*/*z* 302.0932 [M + H]^+^ (calcd. for C_17_H_17_ClNO_2_^+^: 302.0932) [24].

### 2.2. The Pharmacokinetics, Drug-Likeness, and Medicinal Chemistry Friendliness of TQFL12

**Figure 2:**
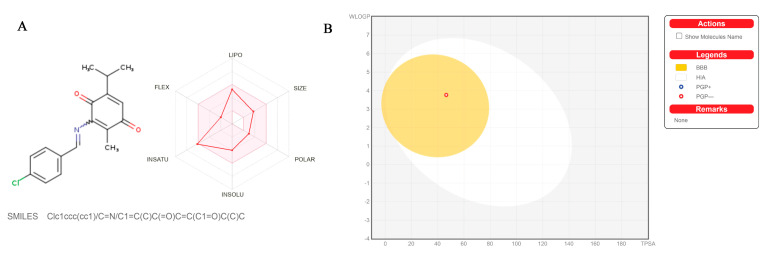
The Bioavailability Radar plot and BOILED-Egg of TQFL12 obtained by using Swiss ADME. (**A**) The 2D chemical structure of TQFL12 (left side) and the Bioavailability Radar plot show the drug-likeness of TQFL12 by five parameters. The pink area represents the optimal range for each property. TQFL12 was predicted to have the potential to become an oral drug. (**B**) The BOILED-Egg was used for evaluation of passive gastrointestinal absorption (HIA) and brain penetration (BBB). The white zone is for the high probability of passive absorption by the gastrointestinal tract, and the yellow zone (yolk) is for the high probability of brain penetration. Yolk and white areas are not mutually exclusive. In addition, the points are colored in blue if predicted as active by P-gp (PGP+) and in red if predicted as non-substrate of P-gp (PGP−). TQFL12 was predicated to a non-substrate of P-gp and was at the position of the double yellow and white areas, which was to be an oral drug. These things considered, the PAINS group which was predicted as the active group in the TQFL12 structure by Swiss ADME was the active quinone group in the anti-cancer effect.

For the purpose of understanding the metabolic changes of TQFL12 in vivo, we predicted various pharmacological data through an online data website (Swiss ADME http://www.swissadme.ch/index.php accessed on 21 June 2023). We can see that TQFL12 was predicted to have a stable drug-like profile by evaluation of five parameters, such as size (150 g/mol < XLOFP3 < 500 g/mol), polarity (−0.7 < XLOFP3 < +5.0), insolubility (−6 < LogS (ESOL) < 0), insaturation (0.25 < Fraction Csp3 < 1), flexibility (0 < Num. rotatable bonds < 9), and lipophility (−0.7 < XLOFP3 < +5.0). In the Bioavailability Radar, the colored zone is suitable physicochemical space, which means that it has the potential to develop as an oral drug (Figure 2A). In the egg plot, we can also see that, with two simple parameters—namely, gastrointestinal absorption (HIA) and blood–brain barrier (BBB) rate—TQFL12 has good metabolic effects as it is not excluded in the two sets of parameters (Figure 2B). The basic properties and pharmacokinetics, drug-likeness, and medical chemical friendliness of TQFL12 are shown in Table 1. It is obvious that the metabolism of TQFL12 in the body was predicted to be very high; TQFL12 can penetrate through the blood and brain barrier and P-gp non-substrate. It can be used as an inhibitor for nearly all liver drug enzymes except for CYP2D6; the bioavailability score is very high at 0.55, which indicated that TQFL12 was predicted to be a highly effective oral drug and have good effects in the body. In terms of drug-likeness, TQFL12 met five scoring criteria, and was predicted to have the potential to become an oral drug (Table 1). As we talked about the design and synthesis of TQFL12 in the above result, we retained the active quinone group of TQ in the structure which played an important role in the anti-cancer effects [25]. We can also see that the PAINS group, which was predicted as the active group in the TQFL12 structure by Swiss ADME, was the active quinone group. It is the most vital structure in the TQ; as a result, we focused on the position 6 to explore the effect of different substituents on their activity. In the previous study, we successfully demonstrated that the added group in position 6 not only improved the activity in the TNBC but also declined the toxicity of TQ in the TNBC cells, which can be safer to use in cancer cells [24].

### 2.3. The Acute Toxicity of TQFL12 in Mice Is Significantly Lower Than That of TQ

After injection for 2 h, mice in the TQ group suffered a series of signs of toxicity including different degrees of decreased activity, unsteady walking, and strong abdominal muscle contraction, i.e., hyperspasmia. At 4 h, hunchback and dyspnea were observed. The main symptoms above-mentioned were gradually recovered at 9 h; then, hair confusion and decreased diet were shown in the mice. Other manifestations were not observed, and, even if the main toxic symptoms recovered after 24 h, all of them died within 48 h. The numbers of dead mice per group were 2, 7, 7, and 8, respectively, on the first day. The mortality rates of each dose group were calculated to be 0, 25%, 87.5%, 87.5%, and 100% in a concentration-dependent manner within 24 h (Table 2). Interestingly, there were milder poisoning symptoms in the TQFL12 group than in TQ; we did not observe the above severe signs of poisoning in the TQFL12 group, and even in the high-dose group not all of them died within one week (Table 2). The numbers of dead mice per group were 3, 6, 8, and 9, respectively, on the 7th day. We thus obtained the mortality of the TQFL12 group in 7 days which the mortality increased in a concentration-dependent manner and were 0, 30%, 60%, 80%, and 90%, respectively (Table 2). Then, the 24 h median lethal dose of TQ was calculated as 33.758 mg/kg (17.5–46.5, 95% confidence intervals). Due to the lack of death data on the first day (no death), the 7th-day median lethal dose of TQFL12 was calculated to be 81.405 mg/kg (31.56–123.40, 95% confidence intervals) (Table 2). Thus, we can conclude that the acute toxicity of TQFL12 in mice is obviously lower than that of TQ.

### 2.4. TQFL12 Did Not Significantly Damage the Important Internal Organs of Mice

**Figure 3:**
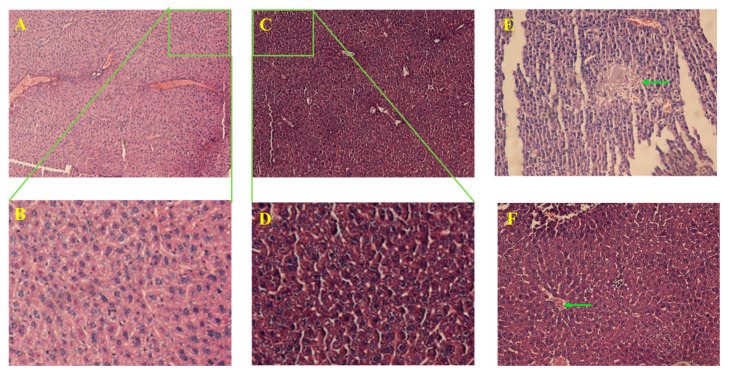
The representative hematoxylin and eosin (H&E) staining images for liver tissues of mice with the treatments of high dose concentration of the indicated drugs. (**A**,**B**) Normal control liver tissues without drug treatments; (**C**,**D**) TQFL12-treated group with unobvious damages in the liver; (**E**,**F**) TQ-treated group. A little focal necrosis and inflammatory cell infiltration occurred in the liver tissues, as the arrow points to. (**B**,**D**) are the enlarged images from (**A**,**C**), respectively. The fresh tissues (about 100 mg were obtained from mice, three representative samples were selected for each group) were removed from mice, washed in PBS three times, and fixed in 4% paraformaldehyde (>24 h) after dehydration by a gradient of alcohol (70%, 80%, 90%, 95%, 95%, 100%, and 100%), cleared in xylene three times, in the paraffin for 1 h three times, and then the tissues were quickly clamped out and laid flat in an embedded box with melted paraffin, cooled, and placed overnight. On the second day, the wax blocks were fixed on a paraffin microtome at 4 μm thickness and dried for 2 h for H&E staining.

**Figure 4:**
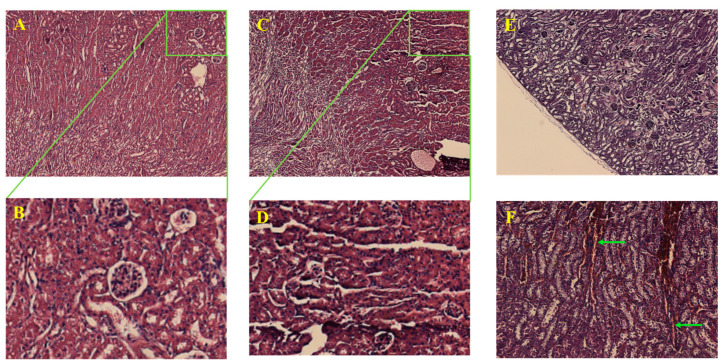
The representative H&E staining images for kidney tissues of mice with the treatments of high dose concentration of the indicated drugs. (**A**,**B**) Normal control kidney tissues without drug treatments; (**C**,**D**) TQFL12-treated group with unobvious damages in the kidney; (**E**,**F**) TQ-treated group with strong renal failure. (**E**) Renal tubular epithelial thinning and interstitial swelling; (**F**) Obvious vascular endothelial swelling, vascular congestion, hemorrhage, and lumen narrowing. (**B**,**D**) are the enlarged images from (**A**,**C**), respectively. The sampling and H&E staining are the same as in Figure 3.

In order to examine the damage in the important internal organs of mice, we obtained the liver and kidney tissues of the high dose of mice, respectively. Firstly, we found that there was just a little focal necrosis and inflammatory cell infiltration in the liver tissues of the TQ group (Figure 3E,F), and most of the liver tissues were not damaged by the drugs. However, the renal tissues of the mice in the TQ group were obviously subjected to severe drug-induced renal failures, such as renal tubular epithelial thinning, interstitial swelling (Figure 4E), and obvious vascular endothelial swelling, vascular congestion, hemorrhage, lumen narrowing (Figure 4F), and so on. Differently, the above-mentioned changes in the TQFL12 group were significantly less severe than those in the TQ group (Figure 3C,D and Figure 4C,D). We hardly found the above severe pathological changes in the TQFL12 group, which indicated that the liver tissues and kidney tissues in the mice were hardly damaged by the TQFL12 drugs, as the tissues of the mice in the negative control group which was normal. Thus, we concluded that, compared with TQ, TQFL12 did not significantly damage the important internal organs of mice, which also indicated lower toxicity than TQ.

### 2.5. TQFL12 Toxicity Resistance in Mice May Be through the AMPKα Signaling Pathway

**Figure 5:**
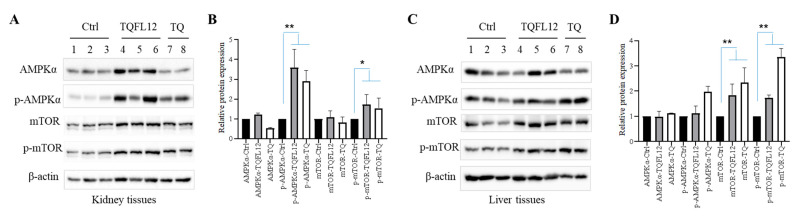
TQFL12 toxicity resistance through the AMPKα signaling pathway. (**A**,**B**) TQFL12-treated group shows up-regulated expression of both total and phosphorylated levels of AMPKα in the kidney tissues and the increase was more than that of the TQ group. (**C**,**D**) TQFL12-treated group shows up-regulated expression of phosphorylated levels of mTOR and the total expression of that in the liver tissues and the increase in the TQ group was more than that of TQFL12. (**B**,**D**) The quantitative data from (**A**,**C**), respectively. * *p* < 0.05, ** *p* < 0.01. The fresh tissues (about 50 mg were obtained from mice, three representative samples were selected from the TQFL12 group and two from the TQ group) from each group were lysed by EBC buffer for 30 min at 4 °C, taken as homogenate, and high-speed centrifugated at 12,000 rpm for 30 min at 4 °C; then, the liquid supernatant proteins samples were extracted and 2× SDS buffer were added and boiled at 100 °C for 5 min. The protein samples were separated in 8% and 10% sodium dodecyl sulfate–PAGE gel, transferred to the polyvinylidene fluoride with 300 mA for 2 h, and the membranes with the methanol were rinsed by TBST. After blocking in 5% nonfat milk, the membranes were incubated with indicated antibodies for Western blotting.

We tried to explore the mechanism by which mice resist TQFL12 toxicity; next, we performed Western blot experiments with tissues of different drug-treated mouse kidney and liver proteins compared with normal mice. The results showed that both TQ and TQFL12 up-regulated the expression of phosphorylated levels of AMPKα in the kidney (Figure 5A), and—compared with TQ, that up-regulated protein levels to nearly 3-fold—the TQFL12 group achieved more up-regulation (Figure 5B), which is in accord with the mild renal failure in the kidney tissues of TQFL12 group (Figure 4C,D). In the liver tissues, TQ and TQFL12 up-regulated the expression of phosphorylated levels of mTOR, and the total expression of that (Figure 5C) and the increase in the TQ group was more than that of TQFL12 (Figure 5D). Interestingly, TQFL12 did not up-regulate the expression of phosphorylated levels of AMPKα as the kidneys showed, which indicated that there was little signaling-pathway activation in the almost-undamaged liver tissues (Figure 3C,D). Thus, we speculate that TQFL12 resists renal toxicity through the AMPK signaling pathway in mice, and we are still exploring more downstream target signaling molecules.

## 3. Materials and Methods

### 3.1. Chemicals and Reagents

TQ was purchased from Sigma-Aldrich (St. Louis, MO, USA). TQFL12 was synthesized by the authors. Dimethyl sulfoxide (DMSO Sigma Cat#SHBL8921) was used to dissolve the drugs. The 4% paraformaldehyde was purchased from Beyotime Biotech Biotechnology Co., Ltd (Shanghai, China). The hematoxylin and eosin (H&E) kit was purchased from Solarbio Technology Co., Ltd (Beijing, China). Ethyl alcohol (EtOH) was obtained from Chuandong Chemical Co., Ltd. (Chongqing, China). Neutral gum (Solarbio Cat#G8590) was used to cover the slice. EBC buffer (0.5% NP-40, 20 mM Tris-HCl pH 8.0, 2 mM EDTA, and 125 mM NaCl; protease inhibitor was added before use) and 2× SDS buffer (60 mM Tris-HCl (pH 6.8), 8% SDS, 0.004% Bromophenol blue, 20% Glycerin, 2% β-Mercaptoethanol) were used for tissue lysis. Methanol was purchased from Chengdu Kelon Technology Co., Ltd. (Chengdu, China). Tris, Glycine, Acrylic amide, β-Mercaptoethanol, Glycerin, SDS, Bromophenol blue, APS, and TEMED were all purchased from BIO-RAD (Hercules, CA, USA). TBST with 0.1% Tween 20 (Chengdu Kelon Cat#9005-64-5) was used to dilute antibodies. The antibodies were mostly purchased from Cell Signaling Technology (Boston, MA, USA) against 5-adenosine monophosphate-activated protein kinase (AMPKα (Cell Signaling Cat#2532)), phosphor-AMPKα (Cell Signaling Thr172, Cat#2535), mammalian target of rapamycin (mTOR (Cell Signaling Cat#2972)), phosphor-mTOR (Cell Signaling Cat#2974), β-actin (Sigma Cat#A1978), anti-rabbit secondary antibodies (Cell Signaling Cat#7074), and anti-mouse secondary antibodies (Cell Signaling Cat#7076). 

### 3.2. Animals

This animal experiment was approved by the Southwest Medical University Animal Ethics Committee (No.: 20210930-007, date: 30 September 2021) and is in strict accordance with the animal welfare protocol [15,24]. BALB/c mice (6~7 weeks old, 18 g) were purchased from Tengxin Biotechnology Co., Ltd. (Chongqing, China). A total of four mice each were kept in a cage of specific pathogen-free conditions with constant temperature (22–24 °C) and humidity (40–60%) environment. A 12 h light/dark cycle was maintained every day, and adequate food and water were provided. After a week, 8-week-old mice (about 20 g) were selected to use for the next experiments.

### 3.3. Pharmacokinetics and Toxicity Estimation

The Swiss ADME (http://www.swissadme.ch/index.php accessed on 21 June 2023) web was used to predict the physicochemical characteristics, pharmacokinetics, drug-likeness, and medicinal chemistry friendliness of TQFL12. By importing the 2D chemical structure of TQFL12 in the square of the web, the SMILE list on the right side was obtained to run and compute. Then, the Pharmacological parameters were listed at the bottom of the web [26].

### 3.4. Acute Non-Specific Toxicity Assays

The mice were divided into two groups randomly, half male and half female; 10 in the experimental group were intraperitoneally (ip) given 5 concentration gradients of TQFL12 with 0, 50 mg/kg, 100 mg/kg, 200 mg/kg, 250 mg/kg; 8 in the positive control group were given the 5 other gradients doses of TQ with 0, 25 mg /kg, 50 mg/kg, 80 mg/kg, 120 mg/kg single intraperitoneal injection, respectively. Each mouse was injected with an average of 0.1 mL of drug solution in DMSO. On the first day of injection, the general health and death of the mice were observed and recorded at 2 h, 6 h, 9 h, and 24 h, respectively. While the animals received the drugs, the general poisoning symptoms were checked, such as animal appearance, activity, mental conditions, dietary conditions, secrets, excretions, body weight, breathing, whether the animals showed abdominal muscle contraction, hyperspasmia, emesis, and whether the toxic symptoms recovered within 24 h. After 24 h, the number of deaths in the TQ group was counted in each concentration. Subsequent data in the TQFL12 group were collected every 24 h.

### 3.5. Lethal Dose for 50% (LD_50_)

The Bliss method was used as follows: After the experiment, convert the dose log x and the mortality probability unit y, and then conduct the regression analysis for x and y, and find the regression equation. The x value when y = 5 is calculated from the regression equation, and, taking the antilog of the x value, the LD_50_ is obtained. The collected daily death data were listed to record the mortality of mice, imported into SPSS software, converted into prohibits, respectively, and then the median lethal doses of TQFL12 and TQ were calculated.

### 3.6. Histology

The liver and kidney tissues of the dead mice in the high-dose group were collected on the fifth day of injection in the TQFL12 group and the second day of injection in the TQ group, respectively; the normal mice were taken for comparison. The fresh tissues (about 100 mg were obtained from each mouse, and three representative samples were selected for each group) were removed from mice, washed in PBS 3 times, and fixed in 4% paraformaldehyde (>24 h) after dehydration by a gradient of alcohol (70%, 80%, 90%, 95%, 95%, 100%, and 100%). After this, they were made transparent in the xylene 3 times, in the paraffin for 1 h 3 times, and the tissues were quickly clamped out and laid flat in an embedded box with melted paraffin, cooled, and placed overnight. On the second day, the wax blocks were fixed on a paraffin microtome at 4 μM thickness and dried for 2 h for hematoxylin and eosin (H&E) staining. The staining was used to stain the tissues to observe whether there were pathological changes and the degree of lesions in the two organs were compared with normal tissues [24,27]. Sections were kept at 62 °C for 2 h, in xylene dewaxed 3 times, gradient alcohol hydration (100%, 100%, 95%, 90%, 85%, and 75%), and they were immersed in hematoxylin dye solution for nucleus staining for 5 min, water-rinsed, differentiated in 1% hydrochloride ethanol for 10 s, water rinsed, then were immersed in eosin dye solution for cytoplasm for 5 min at room temperature, water-rinsed, then dehydrated by alcohol gradient (70%, 80%, 90%, 95%, 95%, 100%, and 100%); neutral gum covers the slice after xylene clearing 3 times. Finally, samples were observed under the microscope (Leica, DM2500).

### 3.7. Western Blotting 

Another part of fresh tissues (about 50 mg obtained from each mouse; three representative samples were selected from the TQFL12 group and two from the TQ group) from each group were lysed by EBC buffer (20 mM Tris-HCl, pH 8.0, 125 mM NaCl, 2 mM EDTA, 0.5% NP-40, protease inhibitors) for 30 min at 4 °C. The homogenate was taken and high-speed centrifugated at 12,000 rpm for 30 min at 4 °C; then, the liquid supernatant protein samples were extracted and 2× SDS buffer was added at 100 °C for 5 min. The sample (about 50 μg) from each mouse was separated into 8% and 10% sodium dodecyl sulfate–PAGE with a voltage of 100 V for 2 h, and transferred to the polyvinylidene fluoride with a current of 300 mA for 2 h. The membranes with methanol were rinsed by using TBST. After blocking in 5% nonfat milk in TBST, they were incubated with the indicated primary antibodies in 2% nonfat milk in TBST at 4 °C overnight. On the second day, the membranes were washed by TBST 3 times and secondary antibodies were kept at room temperature for 2 h and then washed again by TBST 3 times. The antibodies against AMPKα (1:5000 dilution), p-AMPKα (1:5000 dilution), mTOR (1:5000 dilution), p-mTOR (1:5000 dilution), β-actin (1:5000 dilution), anti-rabbit secondary antibodies (1:5000 dilution), and anti-mouse secondary antibodies (1:5000 dilution) were used for incubation. The intensity of each band on the membranes was monitored with an image scanner (Gene Company Limited, Gbox Chemi, DRXV4/1068, Hong Kong, China) with Super Signal West Femto Maximum Sensitivity Substrate (Thermo Fish Scientific, XG346245, Boston, MA, USA) and BeyoECL Plus (P0018S, Shanghai, China).

### 3.8. Statistical Analysis 

The statistical difference analysis was performed by one-way ANOVA using GraphPad Prism 8. A *p* value < 0.05 was considered significant. ** *p* < 0.01 and *** *p* < 0.001 indicate more confidence with *p* values.

## 4. Discussion

This study aimed to examine the acute toxic effects of a novel derivative of TQ, TQFL12, in mice, using TQ as a positive control. As a widely studied natural-product-derived small-molecule compound, previous studies have demonstrated that TQ has low toxicity and showed the safety of its use in experimental animals [28,29]. The researchers found that it does not change the biochemical indicators after oral and intraperitoneal in mice [29,30], but there are still many differences about the non-specific toxic effects of TQ in the reports. El-Dakhakhani studied the LD_50_ of TQ to be 10 mg/kg, and in other research the LD_50_ of TQ was reported to be 90.3 mg/kg and 104.7 mg/kg in mice [29,30]. Similarly, most reported that hardly significant deleterious effects were observed in the mice and rats intraperitoneally injected with doses between 5–12.5 mg/kg of TQ. As a novel derivative of TQ, which is more effective than TQ in treating triple-negative breast cancer, we sought to understand TQFL12’s intuitive toxic effect in vivo and whether it is safer compared to TQ so as to develop a more low-toxicity and high-efficiency molecular anti-cancer drug.

In this study, the LD_50_ of TQFL12 in mice was measured to be 81.41 mg/kg in 7 days. Compared with that of TQ, i.e., 33.75 mg/kg within 24 h, the toxicity of TQFL12 was significantly reduced. Moreover, it could be safer to use. On the other hand, compared with the conclusions of most other researchers, we obtained a lower LD_50_ of TQ, which may be related to the use of DMSO, the solvent of the drug, with a certain additional toxicity, owing to the low solubility in the water of TQ. All in all, we obtained a higher value of LD_50_ of TQFL12 under the same conditions, which significantly indicated that TQFL12 was safer to use in cancer cells and animals than TQ, especially for TNBC. 

In addition, we found that the pathological changes in the kidneys were significantly stronger than those in the livers. Except for a little part of mice in the TQ group where a small amount of focal necrosis in the liver can be found, little obvious changes were observed, which indicates that TQ and its derivatives are mainly metabolized by the kidneys in the body, resulting in certain renal damage; similarly, there was little signaling-pathway activity in the liver tissues, which was in accord with the change in AMPKα and p-AMPKα.

AMPK is a protein kinase that all exists in a heterotrimeric complex from different species, including an α-catalytic subunit, a β-regulatory subunit, and a γ-regulatory subunit composition. The N-terminal of the α-subunit contains a conserved Ser/Thr kinase region. The phosphorylation of the site is required for its kinase activity. It is a cellular energy sensor that plays a key role in regulating energy hemostasis and cellular metabolism, including through phosphorylated acetyl-CoA carboxylase (ACC) [24,31] and hormone-sensitive lipase (LIPE) enzymes synthesizing fatty acids and cholesterol, mTOR, and so on, which may be the important target for varieties of diseases including cancer. [32,33]. In the stress state, AMPK was always activated by metabolic stresses that inhibited cells’ ATP production (hypoxia, hypoglycemia) or accelerated ATP consumption (e.g., muscle contraction). Once activated by stresses, AMPK inhibited the growth of cells by increasing the production of ATP and reducing its consumption, thus achieving the role of protecting the healthy state of the body. In addition to this, AMPK is reportedly the main target for the widely used antidiabetic drug metformin [32]. In our study, when mice received the different drugs, they made different reactions and behaviors, maybe as the AMPK was activated to varying degrees. Mice injected with TQFL12 were protected more well than TQ by more up-regulation of AMPK. In previous studies, we also found that AMPKα is the anti-tumor target of TQFL12 in TNBC [24]. Cancer cells have different metabolic changes compared with normal cells; AMPK may also abolish tumor formation by regulating cell growth, proliferation, autophagy, stress response, and so on. For example, AMPK can phosphorylate p53 and make the protein obtain the activity, thus inducing the cell cycle in the G1/S phase of the cell cycle and inhibiting the growth of the tumors. Activation of AMPK is considered a promising target for tumor therapy [34]. In our toxicity experiments, AMPKα may be also the target of TQFL12. Next, we detected AMPK’s downstream signaling proteins ACC, mTOR, etc. For more AMPKα downstream target molecules, we are still continuing experimental exploration.

We successfully designed a series of experiments to verify the low toxicity of the compound TQFL12 and stimulate AMPK up-regulation, which is considered to be a promising novel anti-cancer compound. In our previous study, TQFL12 was demonstrated to suppress the TNBC cells and anti-cancer in the 4T1 cell xenograft mouse model. In addition, we also found that the mechanism of its anti-cancer activity may be by altering cell apoptosis but slightly affects the cell cycle by conducting flow cytometry in 4T1 and MDA-MB-231. Furthermore, we explored the target molecules of TQFL12 in the TNBC cells; AMPK was affected by the TQFL12 stimulation in TNBC cells. Accordingly, p-ACC, an AMPK downstream target molecule, was also found to be up-regulated. We performed different times of TQFL12 treatment and found that TQFL12 up-regulated the protein levels of p-AMPK and p-ACC in a time-dependent manner but did not affect the mRNA transcription; thus, the cycloheximide (CHX, an inhibitor of protein synthesis) treatment with or without TQFL12 was performed to demonstrate that TQFL12 stabilized AMPK. As a result, we successfully demonstrated that TQFL12 affects the AMPK signaling pathway and stabilizes itself in TNBC cancer cells. In addition, TQFL12 was verified to interact with the hydrophobic surface of AMPKα by conducting molecular docking [24]. Altogether, we designed a series of experiments to demonstrate the suppression of TQFL12 in TNBC cells and clarify the mechanism of its anti-cancer properties. We are now exploring for more effects of TQFL12 in the tumors. According to the useful effect which has been demonstrated of TQFL12 in the TNBC and the more possible effect in other tumors, we thus conducted toxicity research, which may be meaningful for developing a higher-effect and lower-toxicity small-molecule compound drug for tumor patients. 

Small-molecule compounds from natural products are considered to be an effective therapeutic strategy for TNBC. The goal of researchers is to develop promising small-molecule compound drugs. TQ has been demonstrated to be effective for TNBC, but the high toxicity limits the performance of its effects. We successfully found that TQFL12 was more effective and lower toxicity than TQ in TNBC cells. Hence, TQFL12 could be a candidate compound for the therapy of TNBC and has potential clinical values.

## 5. Conclusions

In summary, we designed, synthesized, and compared TQFL12 with TQ, and conducted acute toxicity assays on TQFL12. The acute toxicity of TQFL12 in vivo is significantly lower than that of TQ, and its anti-toxicity mechanism may be through the AMPKα signaling pathway. Thus, given the higher anti-tumor effect on breast cancer and lower toxicity, TQFL12 is expected to be a good prospect for drug development in TNBC patients.

## Figures and Tables

**Table 1:** The pharmacokinetics, drug-likeness, and medicinal chemistry friendliness of TQFL12.

Pharmacokinetics		Drug-Likeness		Medicinal Chemistry	
GI absorption	High	Lipinski	Yes	PAINS	1
BBB permeant	Yes	Ghose	Yes	Brenk	2
P-gp substrate	No	Veber	Yes	Lead-likeness	1
CYP1A2 inhibitor	Yes	Egan	Yes	Synthetic Accessibility	3.29
CYP2C19 inhibitor	Yes	Muegge	Yes		
CYP2C9 inhibitor	Yes	Bioavailability Score	0.55		
CYP2D6 inhibitor	No				
CYP3A4 inhibitor	Yes				

**Table 2:** Results of the lethal doses for TQ and TQFL12 treatments for mice.

	Time (h)	24	48	72	96	120	144	168
TQ	120	8	8	/	/	/	/	/
(mg/kg)	80	7	8	/	/	/	/	/
	50	7	8	/	/	/	/	/
	25	2	8	/	/	/	/	/
	LD50	33.75						
	95%IC	17.5–46.5						
TQFL12	250	1	6	7	7	8	9	9
(mg/kg)	200	2	7	8	8	8	8	8
	100	1	3	4	5	6	6	6
	50	0	1	2	2	2	3	3
	LD50							81.41
	95%IC							31.56–123.40

Note: each control group contains 8 mice and each experimental group contains 10 mice. IC—interval confidence.

## Data Availability

Not applicable.

## Sample Availability

Not available.
